# A Chrismas Prayer

**Published:** 1916-12

**Authors:** 


MRS. F. E. DANIEL, Publisher and Managing Editor.

### A Chrismas Prayer.

Oh, Father, my Father and the Father of all those about me, the unfortunate as well as the fortunate, the sick as well as the healthy, the sorrowing as well as the happy, at this Yuletide turn my heart and the hearts of all Thy children to Thee as never before. May the whole world realize, as it has never yet realized, Thy Fatherhood and the brotherhood of all Thy children; may all feel that since Thou art Love and Goodness, and all are created in Thy image, goodness and love and mercy should abound everywhere and among all peoples.

May the hand of every man who would do evil to his brother man be stayed; may every heart that has wronged another be repentant. May men lift each other up, and not strike down; may the Christ spirit of service to humanity be found everywhere.

May wars cease and peace reign; may strife among men give way to Christ's teachings and His example.

Since I am but one of the least of Thy children, oh, Father! and cannot control nations, help me to so control myself and to so live with those about me that I may exemplify in some small degree the life of Thy Son who came into the world that I might become like Him; and may I ever have the strength of mind and of soul to do my humble part in helpful service to the brethren.

